# Investigation on thermodynamics of ion-slicing of GaN and heterogeneously integrating high-quality GaN films on CMOS compatible Si(100) substrates

**DOI:** 10.1038/s41598-017-15094-1

**Published:** 2017-11-08

**Authors:** Kai Huang, Qi Jia, Tiangui You, Runchun Zhang, Jiajie Lin, Shibin Zhang, Min Zhou, Bo Zhang, Wenjie Yu, Xin Ou, Xi Wang

**Affiliations:** 1 State Key Laboratory of Functional Materials for Informatics, Shanghai Institute of Microsystem and Information Technology, Chinese Academy of Sciences, Shanghai, 200050 China; 2 University of Chinese Academy of Sciences, Beijing, 100049 China

## Abstract

Die-to-wafer heterogeneous integration of single-crystalline GaN film with CMOS compatible Si(100) substrate using the ion-cutting technique has been demonstrated. The thermodynamics of GaN surface blistering is *in-situ* investigated via a thermal-stage optical microscopy, which indicates that the large activation energy (2.5 eV) and low H ions utilization ratio (~6%) might result in the extremely high H fluence required for the ion-slicing of GaN. The crystalline quality, surface topography and the microstructure of the GaN films are characterized in detail. The full width at half maximum (FWHM) for GaN (002) X-ray rocking curves is as low as 163 arcsec, corresponding to a density of threading dislocation of 5 × 10^7^ cm^−2^. Different evolution of the implantation-induced damage was observed and a relationship between the damage evolution and implantation-induced damage is demonstrated. This work would be beneficial to understand the mechanism of ion-slicing of GaN and to provide a platform for the hybrid integration of GaN devices with standard Si CMOS process.

## Introduction

In recent years, due to its direct and wide bandgap, high breakdown voltage, and optical nonlinearity, GaN has attracted much attention for the applications of electronic and optoelectronic devices such as high electron mobility transistors (HEMTs), laser diodes (LDs), light emitter diodes (LEDs) as well as GaN nanophotonics^1–4^. Heterogeneously integrating GaN with Si provides a hybrid platform to realize the integration of GaN high-frequency and high-power electronic devices with Si ICs (integrated circuits) as well as the monolithic on-chip light sources and nonlinear interactions for Si photonics^5–8^. To realize GaN/Si heterogeneous structure, GaN films are usually grown on the Si substrates using metalorganic chemical vapor deposition (MOCVD)^6,9^. However, there are several technological hurdles need to be overcome. For the heteroepitaxy growth of wurtzite GaN film, Si(111) substrate is favored for the epitaxy lattice matching instead of the widely-used Si(100) wafer in the standard IC platform. In addition, the large lattice mismatch between GaN and Si (~17%) causes a high density (typically in the range of 10^9^–10^10^ cm^−2^) of threading dislocations even though several micrometers thick buffer layers are used to annihilate the threading dislocations in the active layer^10^. Therefore, many efforts are needed to integrate high-quality GaN on the Si(100) substrates^11,12^.

The ion-cutting technique, which has been applied for the mass production of SOI (silicon-on-insulator) wafers, appears to be a potential solution by transferring high-quality single-crystalline GaN films on Si(100) substrates^13^. As the GaN films are transferred from the bulk GaN wafer, the density of the threading dislocations is inherited from the bulk GaN, and the lattice mismatch with the substrate does not affect the bonding of GaN films with the handle substrates^14^. Thus, high-quality GaN films can be directly transferred on Si(100) substrates coated by SiO_2_ layer in which the SiO_2_ layer acts as an electrical insulator or optical cladding layer.

The ion-cutting technique combines the ion-slicing and wafer bonding processes^13^. The ion-slicing of GaN has been reported. Wafer-scale GaN films have been transferred on sapphire substrates, on which the homoepitaxial growth of GaN active layer and the fabrication of LEDs have been demonstrated^14–17^. It is widely accepted that the surface blistering of GaN is sensitive to the ion implantation fluence and temperature as well as the annealing conditions^18–20^. Fourier transform infrared spectroscopy indicates that N-H and V_Ga_-H_n_ complexes can be formed due to the combination of the implanted H ions with the N atoms and Ga vacancies (V_Ga_)^21^. The activation energy for GaN surface blistering has been calculated to investigate the detrapping and diffusion of H_2_ in GaN^22^. The ion-slicing of GaN requires much higher H fluence than that of Si. The large H fluence causes large wafer bowing and an external flatting process is needed for the subsequent wafer bonding process^16^. O. Moutanabbir *et al*. demonstrated that the double-side implantation is effective to avoid implantation-induced bowing, and it’s a cost-effective method to transfer two GaN films simultaneously^23^. On the other hand, the extremely high H fluence decreases the quality of GaN films. Therefore, reducing the H fluence is a key issue for the ion-cutting of GaN. However, the underlying physical mechanism of the ion-slicing of GaN with a high H fluence was seldom investigated. In addition to transferring GaN film on sapphire substrates, the heterogeneous integration of high-quality crystalline GaN films with CMOS compatible Si(100) substrates by the ion-cutting technique is also highly required. A. Usenko has claimed a patent on the bonding of GaN and Si-based substrates, but no scientific result of transferring GaN film on Si was reported^24^. The die-to-wafer technique is able to integrate GaN film to the targeted position of large Si wafers using the aligned bonding technique, which has been used in the hybrid integration of III-V semiconductors and Si^25,26^.

In this work, the surface blistering of H-implanted GaN was *in-situ* observed using a thermal-stage optical microscope, and the thermodynamics of GaN blistering was analyzed. Single crystalline GaN films with a size of 10 mm × 10 mm were integrated on CMOS compatible Si(100) substrates using the ion-cutting technique, which was realistic for the die-to-wafer heterogeneous integration.

**Figure 1:**
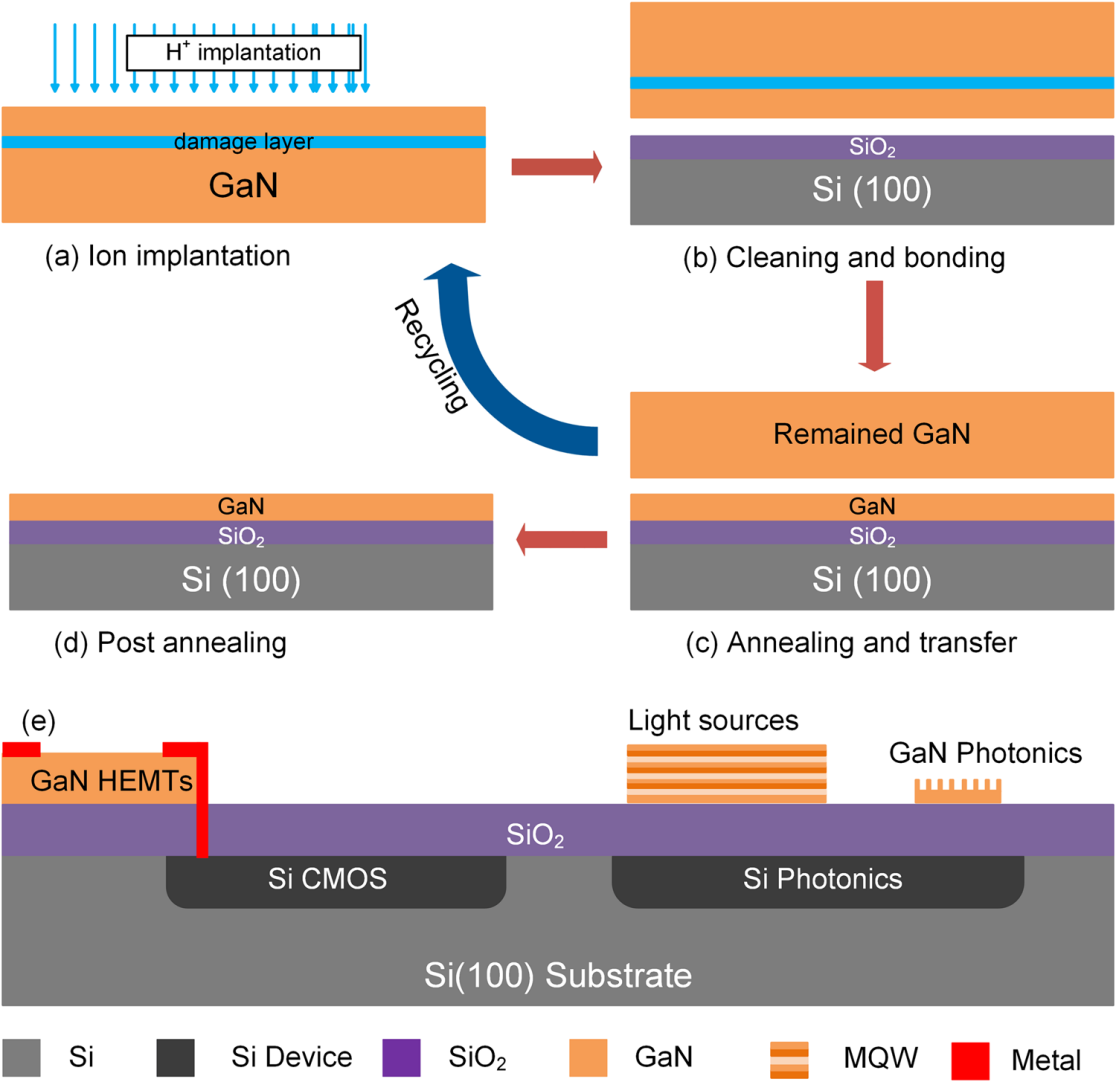
The process flow of the heterogeneous integration of GaN with Si(100) substrates using the ion-cutting method. (**a**) Implanting H ions in the bulk GaN wafer; (**b**) Cleaning and bonding GaN with Si(100) handle wafer; (**c**) Annealing and transferring the GaN film on the Si(100) substrate; (**d**) Post annealing to enhance the bonding strength and to recover the implantation-induced damage. (**e**) Schematic diagram of the die-to-wafer heterogeneous integration of GaN and Si(100) devices.

The process of heterogeneously integrating GaN on Si(100) substrate using the ion-cutting technique is schematically illustrated in Fig. 1. Firstly, a 2-inch bulk GaN wafer purchased from the Hitachi Cable Ltd. was implanted by the 75 keV H ions with a fluence of 3.5 × 10^17^ cm^−2^ at room temperature using a Nissin EXCEED 2300RD ion implanter as shown in Fig. 1(a). During the implantation, the wafer was tilted by 7° from normal to minimize the ion channeling effect. Then, the implanted GaN wafer was diced into small pieces with a size of 10 mm × 10 mm by a diamond knife. Some of the samples were used to investigate the thermodynamics of the ion-slicing of GaN while the others were used to investigate the die-to-wafer bonding process. The surface blistering of H-implanted GaN was *in-situ* observed using a Linkam THMS600 thermal-stage optical microscope (OM). Samples were annealed at different temperatures between 375 °C and 450 °C. Si(100) substrates coated with a thermal SiO_2_ layer were adopted as the handle substrates for the bonding process. After the standard chemical cleaning procedure with RCA solutions and the surface activation with Ar^+^ plasma, the H-implanted GaN samples and Si(100) substrates were bonded at room temperature as shown in Fig. 1(b). As a small GaN substrate was used in the die-to-wafer heterogeneous integration process, the impact of wafer bowing on the die-to-wafer heterogeneous integration was not as significant as on the wafer-scale bonding, and therefore no extra flatten process was performed before the bonding process. After annealing at 550 °C in the N_2_ atmosphere for 2 hours, the GaN film was transferred on the Si(100) substrate and the remained GaN substrate can be reused for the ion-cutting as shown in Fig. 1(c). The transferred GaN film was further post-annealed at 800 °C in the N_2_ atmosphere for 2 hours to enhance the bonding strength and to recover the implantation-induced damage as shown in Fig. 1(d). The die-to-wafer integration technique allows the cooperation of GaN HEMTs and Si CMOS as well as on-chip light source and nonlinear optics for Si photonics as illustrated in Fig. 1(e). The X-ray rocking curves (XRCs) of the (002) and (102) planes of GaN films were recorded using the Philips X’Pert X-ray diffractometer. The non-resonant and resonant Raman spectra of the GaN films were excited by a 514.5 nm and 325 nm laser using the Horiba Scientific LabRAM HR, respectively. The surface topography of GaN films was measured using a Bruker Multimode 8 atomic force microscope (AFM). The microstructure of GaN films and the bonding interface between GaN and SiO_2_ were elucidated by cross-sectional transmission electron microscopy (XTEM) using a JEOL 2100 F field-emission transmission electron microscope.

**Figure 2:**
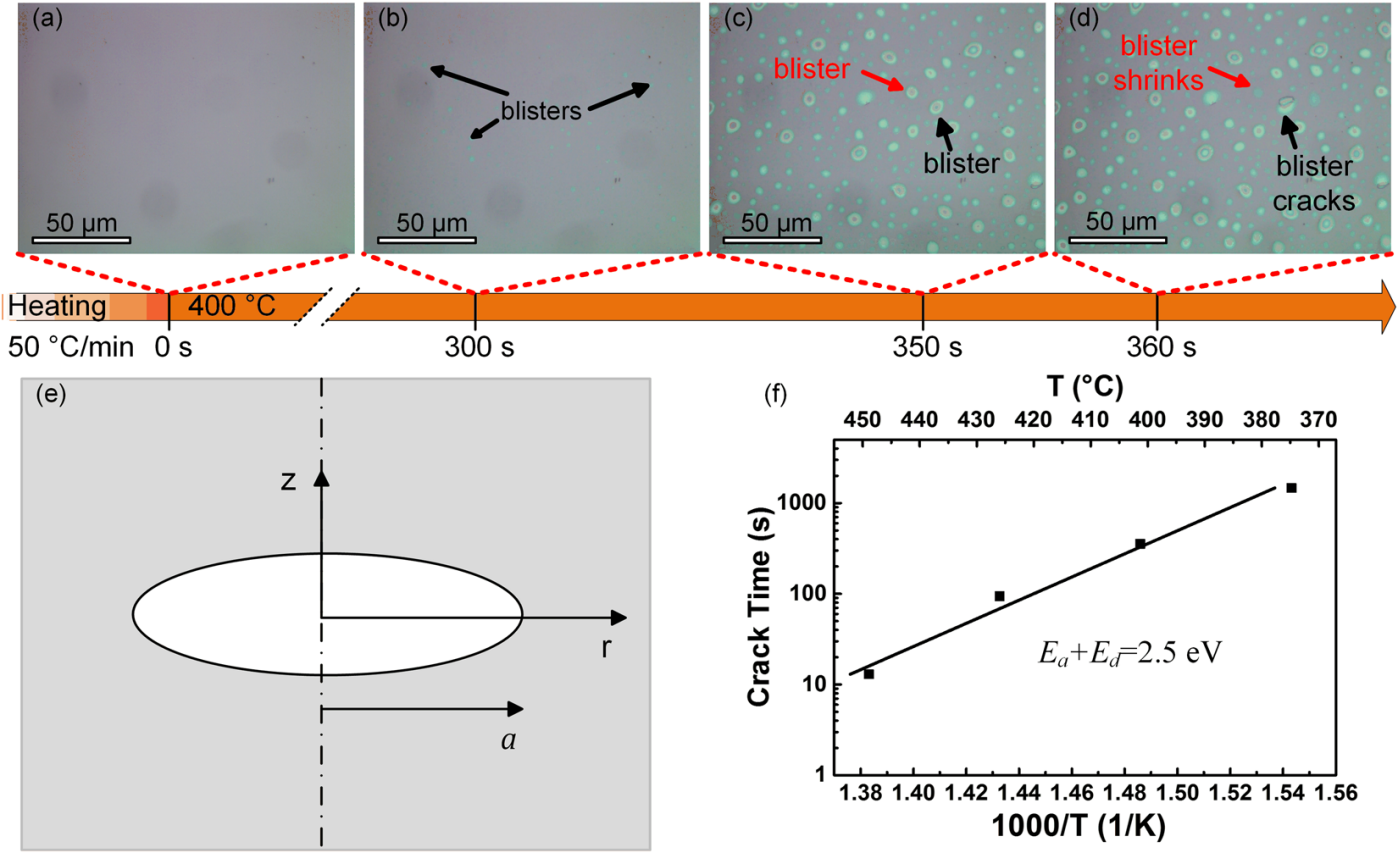
(**a**)–(**d**) *In-situ* OM images of surface topograph variation of H-implanted GaN annealed at 400 °C. The orange line indicates the time shift. (**e**) The schematic diagram of the thermodynamic model. (**f**) Arrhenius plot of the GaN crack time dependent on the annealing temperature. The activation energy of GaN blistering is 2.5 eV.

Understanding the basic mechanism of the crack formation in H-implanted GaN is vital to optimize the ion-slicing process of GaN. Figure 2(a)–(d) show the *in-situ* OM images of the surface morphology variation of H-implanted GaN annealed at 400 °C. There is no change on the surface of H-implanted GaN when the sample is heated to 400 °C. After annealing at 400 °C for 300 s, optically detectable blisters starts to appear on the surface but the incipient blisters are relatively small and sparse as shown in Fig. 2(b). During the annealing, both the blister size and number grow quickly. Figure 2(c) and (d) present two types of blister evolution which were captured at 350 s and 360 s during the annealing at 400 °C. Some blisters grow up continuously and finally crack as marked by the black arrow. In contrast, some blisters grow up at the beginning but shrink as the annealing time increases as marked by the red arrow. A multimedia video of the *in-situ* observation of surface blistering in H-implanted GaN during the annealing process is shown in the Supplementary Video. The thermodynamics of H-implanted GaN was analyzed based on the model for Si proposed by Han *et al*.^27^. This model has been verified by the blistering experiments in Si and Ge, and it was also applied in the investigation of compound materials such as BaTiO_3_ and LiTaO_3_^28–31^. Figure 2(e) shows the schematic diagram of a planar crack with the radius *a*. The radius *a* is determined by the strain energy *U* of bulk GaN around a blister, the crack surface energy *Γ*, and the external potential energy *W*. Thus, the total free energy of a growing hydrogen blister is $G(a,T)=W(a)+U(a)+\Gamma (a,T)$. According to the Griffith energy condition, the criterion for blister growth is taken to be $\partial G(a,T)/\partial a=0$. The critical radius for crack is given as ${a}_{crit}=\pi \gamma (T)E/0.18{p}^{2}(1\,-\,{\upsilon }^{2})$, where *p* is the hydrogen pressure, *E* is Young’s modulus, $\upsilon$ is Poisson ratio, and *γ*(*T*) is the surface tension as a function of annealing temperature *T*. The blister is not thermally stable and will shrink in the case of *a* < *a*_*crit*_, while the blister will grow up and crack in the case of *a* > *a*_*crit*_. The blister shrink and crack are marked by the red arrow and black arrow in Fig. 2 (c) and (d), respectively. When *G*(*a*) = 0, the blister growth will stop and the maximum radius is *a*_*max*_ = 1.5*a*_*crit*_^27^.

During annealing, the H diffused into the blister and increased the initial pressure of the blister. When the initial pressure induced stress exceeds the fracture toughness of GaN, the blisters grow up, accompanied by the pressure decreasing. The trapped H dissociates from N-H and V_Ga_-H_n_ complexes with a binding energy *E*_*a*_ and aggregates with a diffusion energy *E*_*d*_^32^. The reaction rate constant is

1 $$k=\frac{1}{\tau }exp(-\frac{{E}_{a}+{E}_{d}}{{k}_{B}T}),$$

where *T* is the annealing temperature, *τ* is the phenomenological parameter about 10^−14^ s which relates to the stretching frequency of N-H and V_Ga_-H_n_ bond^21^, and *k*_*B*_ is Boltzmann’s constant. The blister radius *a* as a function of annealing temperature *T*, annealing time *t* and H fluence *ϕ*_0_ will be described as^27^

2 $$a(t,T,{\varphi }_{0})=\frac{3\pi E{\varphi }_{0}[1\,-\,exp(-kt)]{k}_{B}T}{16(1\,-\,{\upsilon }^{2}){p}^{2}}.$$

In the formula, the hydrogen pressure *p* is determined by the fracture toughness of GaN and the blister radius^19^. In addition, the relationship between the blistering and the H fluence should be discussed. As there is a fluence window for the ion-slicing of Si and SiC^32,33^, the fluence window might also exist for GaN. Indeed, there is an optimized fluence for the ion-slicing. Below the optimized fluence, the extent of the exfoliation increases with increasing fluence, while it decreases with increasing fluence above the optimized fluence^33^. As the thermodynamic model does not take into account of the negative effect of ultra-high H fluence, it is suitable for the case with the H fluence below the optimized fluence. In our experiment, as the surface blistering was not observed until the fluence increased to 3.5 × 10^17^ cm^−2^, the model is still appropriate.

When the annealing time is long enough, namely $t=\infty$, $a(\infty ,T,{\varnothing }_{0})={a}_{max}$. Since the H fluence ${\varnothing }_{0}$ is 3.5 × 10^17^ cm^−2^ in our experiment, the surface tension is calculated to be $\gamma (T)\approx 1.1\times {10}^{-3}TJ/{m}^{2}$. When the H-implanted GaN cracks, the radius of the growing hydrogen blister should be equal to the critical radius for the crack^27^, namely $a(t,T,{\varphi }_{0})={a}_{crit}$. Therefore, the crack time as a function of the annealing temperature is expressed as

3 $$t(T)=-{10}^{-14}exp(\frac{{E}_{a}+{E}_{d}}{{k}_{B}T})ln(1-\frac{29.6\gamma (T)}{{k}_{B}T{\varphi }_{0}}).$$

The crack time of GaN surface at different annealing temperature was recorded using the *in-situ* thermal-stage optical microscope. Usually, two activation energy values can be extracted from the Arrhenius plot in high-temperature and in low-temperature ranges. In the low-temperature range, H diffusion in GaN is mainly controlled by trapping-detrapping phenomenon, and this might be one of the reasons for the blistering requiring a high H fluence. The Arrhenius plot of the crack time as a function of the annealing temperature *T* below 450 °C is plotted in Fig. 2(f). The activation energy of GaN crack *E*_*a*_+*E*_*d*_ is determined to be 2.5 eV by the slope of the linear fitting. As the blisters appear quickly in the high-temperature range and it’s difficult to record the blistering time precisely, the diffusion energy of free hydrogen in GaN of 0.48 eV recorded by Singh *et al*. was used^22^. Then, the binding energy *E*_*a*_ of N-H and V_Ga_-H_n_ is about 2.02 eV, which is much larger than that of Si (*E*_*a*_+*E*_*d*_ =1.2 eV)^34^. Thus, the trapped H is difficult to be released from the N-H and V_Ga_-H_n_ complexes to generate H_2_ in GaN in comparison to the case of Si^35^. An ideal formula of minimum fluence for surface blistering was given as ${\varnothing }_{min}=\frac{8}{3}\frac{\gamma }{{k}_{B}T}$ by L. B. Freund^36^. By substituting the calculated *γ*(*T*) above in the formula, the minimum H fluence for GaN blistering is estimated to be 2.1 × 10^16^ cm^−2^. However, the surface blistering was not observed experimently until the H fluence was increased to 3.5 × 10^17^ cm^−2^. This means only 6% of the implanted H ions are effective for the surface blistering in our ion-slicing experiment. Most of the implanted H might still be trapped by the N-H and V_Ga_-H_n_ clusters, but only a small portion of implanted H is relased for the H_2_ formation during annealing. The low utilization ratio of implanted H might result in the high H fluence for GaN exfoliation.

For the commercialization, the reduction of the critical fluence for the ion-slicing of GaN would be highly desirable. One of the potential solutions might be the co-implantation of He ions and H ions, which is effective for exfoliation of Si at a reduced fluence^37^. The implanted He ions generate numerous cavity defects which could trap and accumulate the implanted H ions, resulting in the increase of utilization ratio of implanted H and reduction of the critical fluence. A. Tauzin *et al*. suggested that He-H co-implantation could also lower the total fluence required for GaN exfoliation^15^. However, no experimental evidence has been reported so far. Additionally, non-equilibrium thermal process with a high temperature annealing in a subsecond, such as laser annealing and flash annealing, may contribute to release the trapped H in the form of the N-H and V_Ga_-H_n_ complexes, and therefore lower the critical fluence.

**Figure 3:**
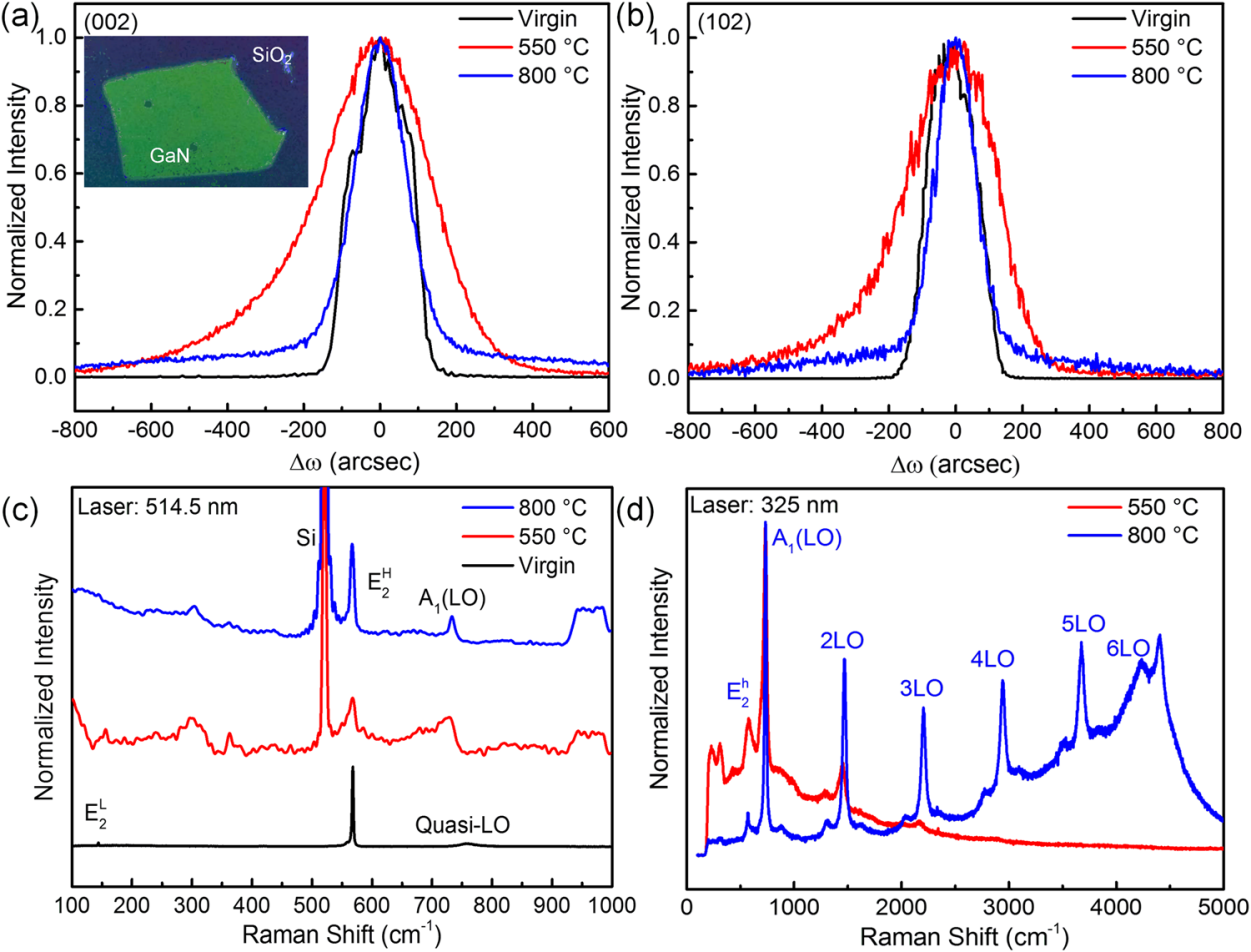
The (**a**) symmetric (002) and (**b**) asymmetric (102) XRCs for the virgin GaN, as-transferred GaN film (transferring at 550 °C) and GaN film post-annealed at 800 °C. The inset in (**a**) shows the photograph of the transferred GaN film. (**c**) Non-resonant and (**d**) resonant Raman spectra of GaN films.

After the bonding and annealing processes, a GaN film with the size of 10 mm × 10 mm was transferred on the Si(100) substrate. The optical photograph of the transferred GaN film is shown in the inset of Fig. 3(a). As the transferred GaN film has a limited size, no cracks was observed on the GaN surface, as indicated by the OM image in Fig. S1 in the Supplementary Information. The crystalline quality of the GaN films was evaluated by XRC measurements to investigate the lattice disorder in the transferred GaN film induced by the high H implantation fluence. The normalized symmetric (002) and asymmetric (102) XRCs for the virgin GaN sample, the as-transferred GaN film (transferring at 550 °C) and the GaN film post-annealed at 800 °C are shown in Fig. 3(a) and (b). The full width at half maximum (FWHM) of the (002) XRC for the virgin sample is 191 arcsec. The FWHM of the as-transferred GaN thin film is 339 arcsec, but it significantly decreases to only 163 arcsec after the post-annealing at 800 °C. The density of the threading dislocations of the GaN film is determined to be as low as 5 × 10^7^cm^−2^ according to the FWHM of the (002) XRC^1,38^. This value is at least one order of magnitude lower than that of the Si-based GaN acquired by the heteroepitaxy using MOCVD^6^. The FWHM of the asymmetric (102) XRCs for the virgin sample and the as-transferred GaN film is 179 arcsec and 318 arcsec, respectively. While the FWHM of the asymmetric (102) XRC is only 140 arcsec for the GaN film after post-annealing at 800 °C. Both the symmetric (002) and asymmetric (102) XRCs are broadened by the implantation-induced damage but narrowed due to the damage recovery after the high-temperature post-annealing. It is worth noting that the FWHM of the post-annealed GaN film is even smaller than that of the virgin sample. It might be due to the recovery of intrinsic defects in GaN during the post-annealing, which can be confirmed by the reduced FWHM of the virgin GaN annealed under the same conditions. The XRCs of the virgin GaN, annealed virgin GaN and the post-annealed transferred GaN film are shown in Fig. S2 and Fig. S3 in the Supplementary Information. The Raman spectra were used to investigate the defect evolution. Figure 3(c) shows the non-resonant Raman spectra of virgin GaN and the transferred GaN films. The typical E_2_^H^ mode (~568 cm^−1^) and the A_1_(LO) mode (~734 cm^−1^) of wurtzite GaN are observed in the transferred GaN films. In comparison with the as-transferred GaN film, the FWHM of the E_2_^H^ mode decreases from 13.6 cm^−1^ to 7.5 cm^−1^ after post-annealing at 800 °C, indicating the improvement of the film quality. In comparison with the Raman spectra of virgin GaN, the peaks observed at 300 cm^−1^ and 360 cm^−1^ in the GaN film are defect-related modes induced by the implantation-induced damage. It has been evidenced that implanted H ions induce a damage zone in the near-surface region of the transferred film by the ion-cutting technique^17^. Since GaN is transparent to the visible light, the 514.5 nm laser is able to penetrate the GaN film. In order to investigate the crystal quality of the damage zone in the near surface of GaN films, the resonant Raman spectra excited by the 325 nm laser was used with a penetration depth of about 100 nm in GaN^39^. Figure 3(d) shows the normalized multiphononon resonant Raman scattering spectra of transferred GaN films. For the as-transferred GaN film (transferring at 550 °C), the third-order LO multiphonon scattering is observed and the photoluminescence (PL) signal is quenched due to the implantation-induced damage. After the post annealing at 800 °C, up to sixth-order peaks with a very weak PL signal are presented. The multiple LO modes and PL quenching indicate that the near-surface region of GaN film is heavily damaged. The generation of multiple LO modes in GaN films results from the residual defects. The implantation-induced defects scatter exciton to form bound excitons which result in the LO photon^39^. The higher order Raman scattering might result from the larger non-zero scattering wave vectors dependent effects of the Frӧhlich interaction and defect-induced exciton scattering^40^. The non-resonant and resonant Raman spectra reveal contrary damage evolution in the GaN films. The Raman spectra excited by incident laser with different wavelength reflect the material quality at different depth region of the GaN films. During the high-temperature post-annealing, the implantation-induced damage in the deep region of the GaN films was recovered, while the quality deterioration at the near-surface region might arise from the GaN decomposition.

**Figure 4:**
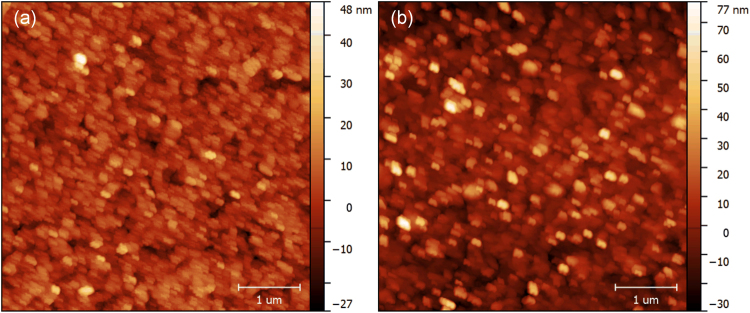
AFM images of (**a**) as-transferred GaN film and (**b**) post-annealed GaN film. The RMS roughness are 6.35 nm and 11.93 nm, respectively.

Figure 4(a) and (b) show the AFM images of the as-transferred GaN film and GaN film post-annealed at 800 °C, respectively. Due to the presence of implantation-induced crack layer^41^, the surface of the as-transferred GaN film is quite rough and the root-mean-square (RMS) roughness in a scanning area of 5 μm × 5 μm is about 6.35 nm. After post-annealing at 800 °C, the RMS roughness increases to 11.93 nm due to the GaN surface decomposition at high temperature. The SEM images of the GaN films and decomposed GaN surface are shown in Fig. S4 in the Supplementary Information. It is suggested that annealing under NH_3_ atmosphere might be helpful to suppress the decomposition of GaN.

**Figure 5:**
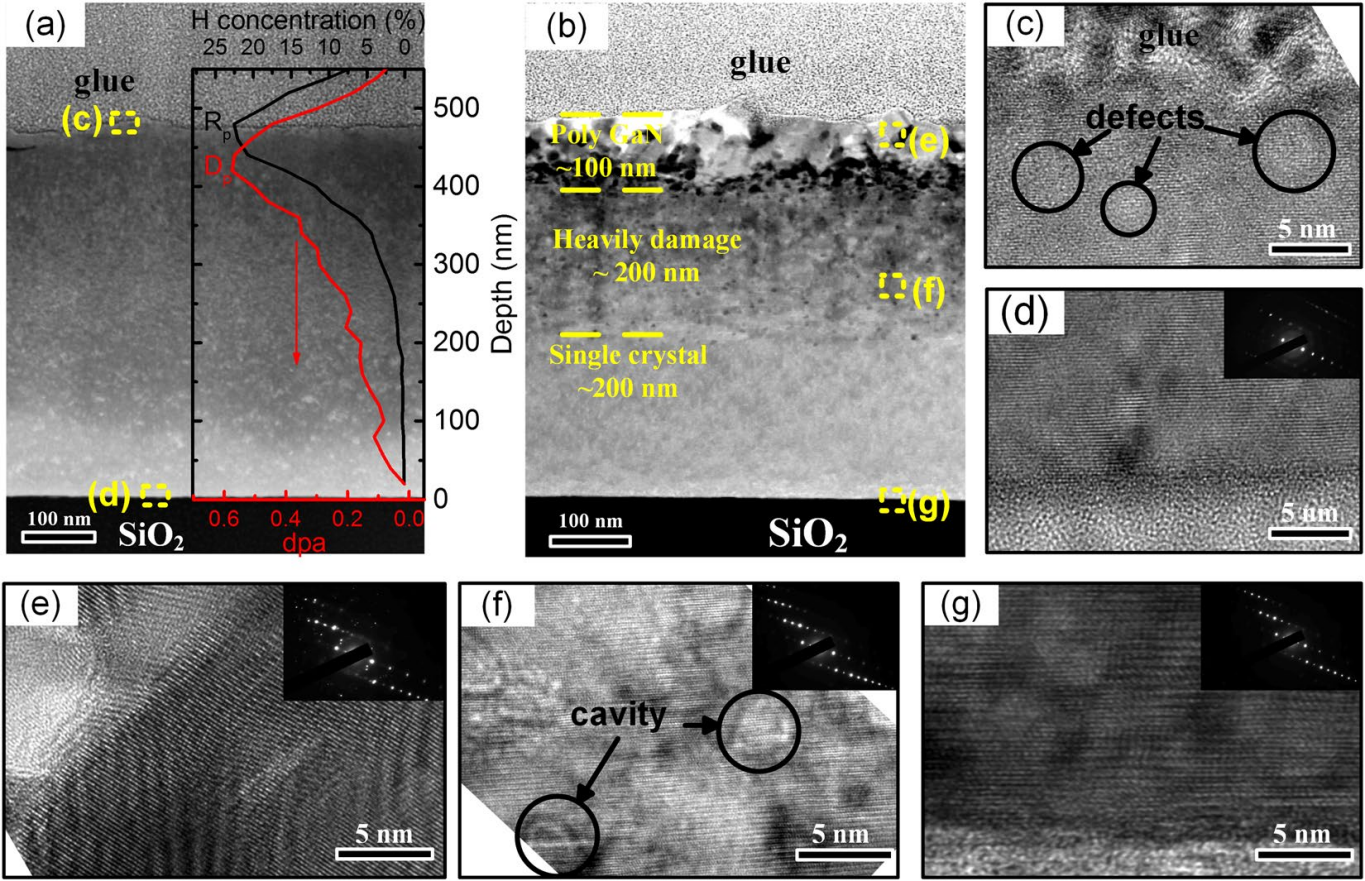
(**a**) XTEM image of the as-transferred GaN film with the simulated H and dpa profiles in the inset. (**b**) XTEM image of the GaN film post-annealed at 800 °C, and three depth regions with different lattice damage are marked by the dash line. (**c**)–(**g**) HRTEM images and SAED patterns taken from the area marked in (**a**) and (**b**).

Figure 5(a) and (b) show the XTEM images of the as-transferred GaN film and GaN film post-annealed at 800 °C, respectively. The thickness of the GaN film is about 480 nm. From the high magnification XTEM images of the as-transferred GaN film shown in Fig. 5(c) and (d), it can be seen that a large number of nano-cavity defects distributed widely in the near-surface region, while the GaN film in the deep region is of single-crystalline quality. The H and damage (displacements per atom, dpa) profiles of SRIM-2013 simulation^42^ are plotted in the inset of Fig. 5(a). The H and damage in GaN are both Gaussian distributed. During annealing, the implanted H ions migrate and accumulate to the peak of the Gaussian distribution of H ions (stopping range R_p_), meanwhile the implantation-induced defects, eg. vacancies, vacancy clusters and bubbles, grow up due to the Ostwald ripening thermal growth (as shown in the Supplementary Video), resulting in the GaN film exfoliation near the stopping range R_p_. The nano-defects at the near-surface region in Fig. 5(c) are residual defects after the film transferring. In comparison with the XTEM image of the as-transferred GaN film shown in Fig. 5(a), three depth regions with different lattice damage are observed in the post-annealed GaN film as shown in Fig. 5(b), i.e. the poly GaN region, the heavily damaged GaN region, and the single crystalline GaN region. The residue defects after the film transferring contribute to the formation of different damage regions during the post-annealing. The poly GaN region consisting of GaN grains with various orientations is about 100 nm in thickness. The HRTEM image in Fig. 5(e) shows that the GaN grain contains lattice disorder, corresponding to unregular diffraction spot in the selected area electron diffraction (SAED) pattern in the inset. The poly GaN region might be caused by the GaN decomposition at high temperature. It is reported that GaN is thermal stable with a decomposition temperature higher than 1000 °C^43^. However, the large amounts of nano-defects might reduce the threshold temperature of the surface decomposition and recrystallization. The heavily damaged GaN region consists many plateau and cavity defects, and the thickness of this damage region is about 200 nm. The cavity defects are generated by the sequential annealing at 550 °C and 800 °C^44^. The high-temperature post-annealing accelerates the migration and accumulation of the residual nano-defects formed during the GaN transferring at 550 °C, and hence the cavity defects with large size are formed. From the HRTEM image shown in Fig. 5(f), lattice fringe can be observed in the cavity defects, indicating that the spacing of lattice planes near the cavity was unaffected by the cavity defects. This is confirmed by the SAED pattern in the inset with a regular diffraction pattern. Figure 5(g) is the HRTEM image of the single crystalline GaN region close to the bonding interface. The GaN film and the bonding interface are of high quality. The damage level of the post-annealed GaN film is related to the dpa value. The dpa is larger than 0.4 for the poly GaN region and it is between 0.18 and 0.4 for the heavily damaged GaN region. In the single crystalline GaN region, the dpa is smaller than 0.18 and the residual defects can be recovered during the high-temperature annealing. As the threshold for damage recovery is about 0.18 dpa, lower H fluence is helpful to reduce the thickness of the damaged GaN region. In comparison with the as-transferred GaN film, more defects are visible in the XTEM images but the XRCs indicate a higher quality of the post-annealed GaN film. This is because the cavity defects have a smaller impact on the lattice structure than the sparsely distributed nano-defects, which results in a narrower FWHM of XRCs. The similar results were also reported by Dadwal *et al*. and Hayashi *et al*.^45,46^. After revoming the top damage layer, the ramained GaN film will retain the high quality of the bulk GaN. For the further electronic and optoelectronic active layer epitaxy and device fabrication, chemical mechanical polishing (CMP) process is required to remove the heavily damaged layer and to acquire a smooth surface.

In conclusion, crystalline GaN films have been integrated with CMOS compatible Si(100) substrate coated with SiO_2_ layer by using the ion-cutting technique. For the ion-slicing of GaN, the thermodynamics of H-implanted GaN was *in-situ* investigated via a thermal-stage OM. The activation energy of GaN surface blistering was estimated to be as high as 2.5 eV, and only 6% of the implanted H ions contribute to the blistering, which result in the high H fluence required for ion-slicing of GaN. After post-annealing at 800 °C, the FWHM of the (002) XRCs for the transferred GaN film was only 163 arcsec and the density of threading dislocations was estimated to be 5 × 10^7^ cm^−2^. The thermal evolution of the damages was investigated in detail by non-resonant and resonant Raman spectra and XTEM. The die-to-wafer structure could be exploited for the integration of GaN-based high-frequency and high-power HEMTs with Si ICs, the second harmonic generation for Si photonics as well as on-chip the light source for all optical communication.

## Electronic supplementary material

Surface blistering video

Supplementary Information
