# Supplementary material for: Investigation on thermodynamics of ion-slicing of GaN and heterogeneously integrating high-quality GaN films on CMOS compatible Si(100) substrates
Source: Sci Rep. 2017 Nov 8;7:15017. doi: 10.1038/s41598-017-15094-1 (PMC5678072; doi:10.1038/s41598-017-15094-1)
Supplement: Supplementary file 2 — Supplementary Information [file 41598_2017_15094_MOESM2_ESM.pdf]

Supplementary Information for

Investigation on thermodynamics of ion-slicing of GaN and heterogeneously integrating high-quality GaN films on CMOS compatible Si(100) substrates

Kai Huang,[1,2] Qi Jia,[1,2] Tiangui You,[1*] Runchun Zhang,[1,2] Jiajie Lin,[1,2] Shibin Zhang,[1,2] Min Zhou,[1] Bo Zhang,[1] Wenjie Yu,[1] Xin Ou[1*] and Xi Wang[1]

[1]State Key Laboratory of Functional Materials for Informatics, Shanghai Institute of Microsystem and Information Technology, Chinese Academy of Sciences, Shanghai 200050, China

[2]University of Chinese Academy of Sciences, Beijing 100049, China

[*] E-mail: t.you@mail.sim.ac.cn (Tiangui You)   ouxin@mail.sim.ac.cn (Xin Ou)

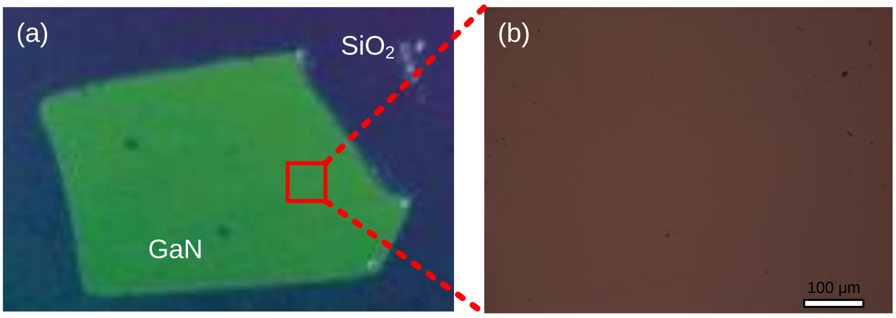

Fig. S1 OM image of the GaN film surface after high temperature post-annealing.

Fig. S1 shows the OM image of the surface of post-annealed GaN film. As the the die-to-wafer integration method is used to integrate GaN film with the CMOS compatible Si(100) substrate, the small size of GaN film is effective to reduce the thermal stress caused by the coefficient of thermal expansion mismatch between GaN and Si. No cracks are observed on the surface of the transferred GaN film after post-annealing at 800 ℃.

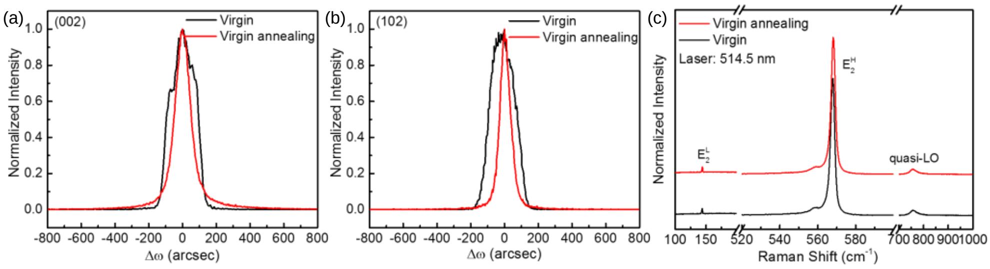

Fig. S2 (a)-(b) XRCs of virgin GaN and GaN after annealing at 800 ℃. (c) Raman spectra of virgin GaN and GaN after annealing at 800 ℃.

Fig. S2 shows the XRCs and Raman spectra of virgin GaN and virgin GaN after annealing at 800 ℃. As shown in Fig. S2 (a) and (b), the FWHM values of the (002) and (102) planes decreased to 108 arcsec and 72 arcsec, respectively. In Fig. S2 (c), the FWHM of the E_2^H shows no obvious change only the FWHM of the quasi-LO mode decreases from 26.5 cm^{-1} to 23 cm^{-1}. The XRCs rather than the Raman spectra indicate the recovery of the intrinsic defects by the post-annealing. X-ray diffraction is more sensitive to detect the changes of intrinsic defects than the Raman spectra. The XRCs of transferred GaN film are broadened by the implantation-induced damage, but

narrowed by the recovery of implantation-induced defects and intrinsic defects after high-temperature post-annealing. The combination effect results in that the FWHM of the post-annealed GaN film is smaller than that of the virgin GaN.

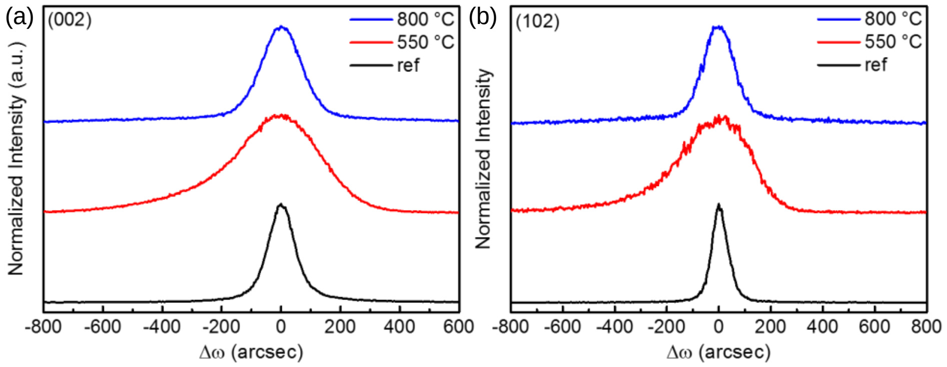

Fig. S3 XRCs of the GaN film in comparison with the annealed virgin GaN (ref).

Fig. S3 shows the XRCs for the annealed virgin GaN, the as-transferred GaN film and the post-annealed GaN film. In comparison with the annealed virgin GaN (ref), the XRCs of the post-annealed GaN film are broadened. The FWHM of the post-annealed GaN film is larger than the annealed virgin GaN but smaller than the virgin GaN. It's a combination effects of the implantation-induced damage, the recovery of implantation damage and the recovery of intrinsic defects.

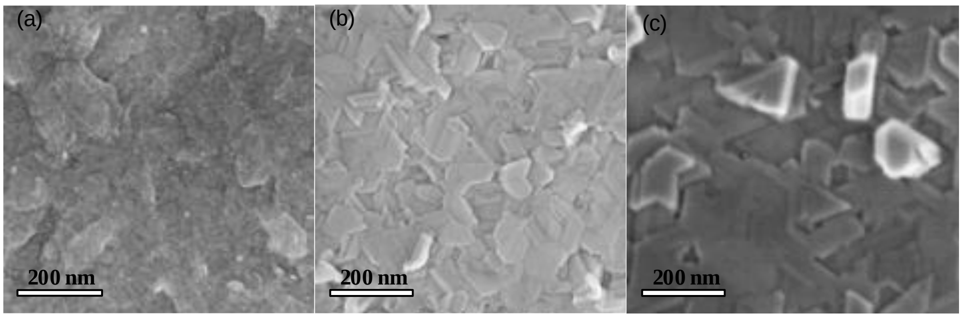

Fig. S4 SEM images of surface morphology of (a) as-transferred GaN film, (b) post-annealed GaN film and (c) decomposed virgin GaN.

As the implantation-induced crack defect layer in the H-implanted GaN is not flat, the surface of the as-transferred GaN film is relatively rough as shown in Fig. S1(a). Fig. S1 (b) and (c) show the surface morphology of the GaN film post-annealed at 800 ℃ and bulk GaN annealed at 1000 ℃, respectively. The surface morphology of

the post-annealed GaN film (Fig. S1 (b)) is similar to that of the decomposed GaN surface (Fig. S1 (c)). The increased roughness of GaN film after high-temperature post-annealing (Fig. 4) is caused by the GaN decomposition at the near-surface region.
